# Change to an Informal Interview Dress Code Improves Residency Applicant Perceptions

**DOI:** 10.5811/westjem.2014.11.22982

**Published:** 2014-12-09

**Authors:** H. Gene Hern, Charlotte P. Wills, Brian Johnson

**Affiliations:** Alameda Health System, Highland Hospital, Department of Emergency Medicine, Oakland, California

## Abstract

**Introduction:**

Residency interview apparel has traditionally been the dark business suit. We changed the interview dress code from a traditionally established unwritten ‘formal’ attire to an explicitly described ‘informal’ attire. We sought to assess if the change in dress code attire changed applicants’ perceptions of the residency program or decreased costs.

**Methods:**

The authors conducted an anonymous survey of applicants applying to one emergency medicine residency program during two application cycles ending in 2012 and 2013. Applicants were asked if the change in dress code affected their perception of the program, comfort level, overall costs and how it affected their rank lists.

**Results:**

We sent the survey to 308 interviewed applicants over two years. Of those, 236 applicants completed the survey for a combined response rate of 76.6% (236/308). Among respondents, 85.1% (200 of 235) stated they appreciated the change; 66.7% (154 of 231) stated the change caused them to worry more about what to wear. Males were more uncomfortable than females due to the lack of uniformity on the interview day (18.5% of males [25/135] vs. 7.4% of females [7/95], collapsed results p-value 0.008). A total of 27.7% (64/231) agreed that the costs were less overall. The change caused 50 of 230 (21.7%) applicants to rank the program higher on their rank list and only one applicant to rank the program lower.

**Conclusion:**

A change to a more informal dress code resulted in more comfort and fewer costs for applicants to a single residency program. The change also resulted in some applicants placing the program higher on their rank order list.

## INTRODUCTION

The interview for residency remains a rite of passage for physicians seeking specialty training throughout the country. Usually during their last year of medical school, students apply to residency programs in the specialty of their choice. During the months of November through January they interview at multiple residency training programs, and then list the programs they prefer in the rank order list (ROL). One tradition of the interview, which has remained across specialties, is the formal business attire. However in recent years, the dress code of medical schools has become more relaxed to even allow fleece jackets or ‘sneakers’.^1^,^2^ It is less common for medical students whether in the classroom, clinic, or wards to be dressed in formal business attire, especially if ‘on call’ or doing a rotation in the emergency department or intensive care unit.^3^

Few articles about dress codes for physicians exist and what does exist does not describe residency interviews. Furthermore, few articles actually study any dress attire phenomena beyond patient perceptions. No study to date has addressed cost or physician perceptions with dress code. What does exist concerns the dress code for medical student or physicians while seeing patients.^4^,^5^ In 2007, the United Kingdom’s National Health Service proposed a ‘Bare Below the Elbows’ dress code for infection control, which was met with some resistance.^6^–^8^

We challenged the dominant paradigm of “a residency interview requires business attire” by explicitly changing the dress code expectations for applicants on their interview day at our emergency medicine (EM) residency training program. We then explored whether changing the dress code for residency interviews also changed applicants’ perceptions of the program, comfort level, or decreased the cost of the interview season. Furthermore, we surveyed applicants about their opinions as to whether EM residency programs should adopt this practice.

## METHODS

During the initial interview offers to applicants of our residency program in the 2011–12 and 2012–13 seasons, we stated in the letter that formal business attire was not required. The letter stated, “We don’t want you to wear suits to the interview day, unless you absolutely love wearing suits. We will all be dressed in some version of jeans, scrubs, and at the most, business casual.” We reiterated this new policy in our follow-up communication and provided examples of appropriate but more casual attire. Once the interviewees had submitted their match list and the program had also submitted the match list, we surveyed applicants who had interviewed at our program using a commercially available survey website (SurveyMonkey.com). The survey was submitted to the institutional review board at our home institution, Alameda Health System, and was approved.

We developed and then piloted our survey with departmental education faculty with a combined total of 40 years of residency leadership experience. In addition, the survey was piloted with residents within our own program (a group generally similar in age to our target population and who went through the interview process recently.) The pilot and revisions were in accordance with survey design methodology to maximize validity and reliability.^9^ No prior studies of this topic exist to adapt survey instrument questions from.

The survey was emailed to residency applicants twice after the deadline to submit rank order lists for both programs and applicants had passed. The option to respond was closed prior to the release of match results to either applicant or program. Respondents were allowed to not answer all of the questions. We surveyed applicants about demographic information including age, gender, and geographic location. In addition, applicants were queried about their impressions of the change in dress code expectations. For an example of the instrument, see attached survey.

We collapsed the five-point Likert scale anchored by “1-disagree” and “5-agree” into 1 to 2 (disagree), 3 (neutral), and 4 to 5 (agree). A general comments section was included at the end of the survey. We performed statistical analysis using SAS (Version 9.3, SAS Inc; Cary, NC). Each survey question was analyzed by gender, age, and medical school region. We calculated differences using chi square analysis for categorical variables (age, medical school region) and t-test for age.

## RESULTS

We sent the survey to all 150 interviewed applicants the first year and all 158 the second. It was completed by 120 applicants the first year and 116 applicants the second year for a combined response rate of 76.6% (236/308) (Table 1 contains respondent demographic information). Most respondents felt comfortable with the change in dress code expectation. Among respondents, 85.1% (200 of 235) stated they appreciated the change to a more casual dress code. The majority of applicants did not feel less comfortable as compared to their experiences at other programs (75.5%, 173/229). Of note, when asked if the change in the dress code caused them to worry more about what to wear, 66.7% (154 of 231) replied ‘yes;’ however, during the interview day, the majority were not uncomfortable by the lack of uniformity in apparel (74.9%, 173/231) (Table 2). There was no difference in response by gender except more males were uncomfortable than female applicants due to the lack of uniformity on the interview day (18.5% of males [25/135] vs. 7.4% of females [7/95], collapsed results p-value 0.008) (Table 3).

Overall, a minority of applicants stated that a change in dress code cost them less money overall in the interview process. Only 27.7% (64/231) agreed that the costs were overall less compared to costs incurred interviewing at other residency training programs.

Regarding perceptions of the residency program itself, 212 of 234 (90.6%) ‘disagreed’ or ‘somewhat disagreed’ with the statement “The change in dress expectation lowered [program] in my esteem.” A confirmatory question later in the survey asked whether the dress code change improved their feelings of the program. Of respondents, 150 of 229 (65.5%) stated the change in dress code improved their feelings about the program and an additional 66 (28.8%) stated it had no change on their feelings about the program. Only six applicants of the 234 (2.6%) ‘somewhat agreed’ that the change in dress code lowered their esteem of the program and two applicants took the stronger position of ‘agreed’ (0.9%). When asked if the change in dress code reflected the attitudes and demeanor of the programs’ attendings and residents, 203 of 234 (86.8%) responded in the affirmative.

Per survey responses, the dress code change during the interview season increased discussion with other applicants also in the residency interview process. When asked if other applicants discussed the dress code change while interviewing at other programs, 195 of 234 (83.3%) stated that other applicants brought up the topic. The question expressly stated that they, the applicant, did not bring up the topic in conversation.

When asked if other residency programs should change their dress code, 148 of 232 (63.8%) stated they agreed or somewhat agreed. Additionally, another 51 (22%) stated they neither agreed nor disagreed. There were no statistical differences by school region or age for those in favor of dress code change. Only 4.2% of applicants from the Southern region (1/25) and one of 13 applicants (7.7%) from the Rocky Mountain region were not in favor of a dress code change. (Perhaps we should explicitly say there were no differences by school region or age.)

The change in dress code caused 50 of 230 (21.7%) applicants to rank the program higher on their rank list. Only one applicant indicated the dress code change caused him or her to rank the program lower. We found no significant difference in response to any dress code question by age or medical school region.

Anecdotally, there were a few applicants (less than 10) who still wore formal business attire to the interview. Applicants who interviewed earlier in the season seemed to have more apprehension with the new dress code than later applicants. It may be that later applicants heard about the dress code from fellow interviewees at other programs or their own medical school and had more time to confirm the information being sent from the program itself.

The authors do not believe there were negative affects on the program itself. The program’s rank list was filled within three spots of prior years’ matches, so it appears that at least for those years’ rank lists, there was no negative impact to the program.

## DISCUSSION

Our experience demonstrated that changing dress code expectations from formal to more casual did not negatively impact the interview process for either applicant or program at our single institution. The results of our survey demonstrate the majority of applicants to our EM residency preferred to have a more casual interview dress code.

This result must be taken in the context of the study. This was a change in dress code in one EM program over two years. While the results suggest relatively little impact upon the program reputation or the ranking of the program by applicant, one must consider the setting.

EM is a relatively newer specialty that does not have a ‘clinic’ type setting, and thus the practitioners tend to wear surgical scrubs in the emergency department. A change in interview attire may not be seen as acceptable to more traditional specialties who retain a clinic or office based setting. These specialists often have more formal attire during these clinical settings.

In terms of cost savings, a change in dress code may not provide dramatic cost savings. Anecdotally, many applicants have commented over the years on the cost of flying all over the country doing interviews. Some have applied for additional credit cards or applied for loans from their medical schools to help defray the costs of interviewing. We had hoped the change in dress code might limit some of the costs of cleaning or maintaining their formal business attire but that may not be the case. Applicants still have to have their formal business attire for interviewing. More money is likely saved by staying overnight with current residents/friends and taking public transit/carpooling (all practices we encourage), rather than hotels and rental cars.

### Limitations

The study suffers from a number of limitations common to surveys. Recall bias is a clear limitation as surveys were completed perhaps more than a month after the actual interview. In addition, despite the anonymous survey being sent in the ‘quiet period’ when the National Resident Matching Program has each program’s and applicant’s list, it is possible that applicants felt their response was perhaps traceable back to them or might negatively influence their application or their ranking.

While the response rate was 76.6%, it is also possible the applicants who did not answer the query had more negative feelings about the dress code change and might have lowered the favorable percentages or have resulted in applicants stating they lowered the program on their rank lists.

Additionally, the residency program where this change in dress code was implemented trains EM residents. This is a specialty that does not have ‘clinic days’ or any standard outpatient office setting. Applicants who prefer this specialty may also tend to prefer acute care settings in which scrubs are more favored. Finally, the program is also located in a county hospital in California and where the ‘formality’ of the hospital and the faculty might be more informal than other institutions located in other parts of the country. As an example, most faculty do not wear white coats and some have tattoos visible.

Another potential limitation is that we did not explicitly notate which applicants did not follow the new dress code and were dressed in a more traditional fashion. We, therefore, could not exclude them from the study as the survey was sent anonymously to all applicants who interviewed.

## CONCLUSION

Changing the dress code for the residency interview does not appear to negatively affect the program’s reputation or rank order list results. Most applicants prefer a less formal dress code. It appears to make them more comfortable and, for a minority, incurs less cost. The change in dress code at the single study site was associated with some applicants raising the program on their rank lists and very few lowering it. Further investigations should study how individual applicants are perceived by their interpretation of the dress code and if some are ranked higher or lower based on their actual apparel or their ‘following’ the suggested dress code.

## Figures and Tables

**Table 1:** Demographics of surveyed applicants with regard to interview dress code (mean age 27.7).

Variable	n (%)
Gender	
Male	137 (58.3)
Female	98 (41.7)
Medical school region	
East Coast	77 (33.5)
Midwest	34 (14.8)
Rocky Mountain	14 (6.1)
South	24 (10.4)
West Coast	81 (35.2)
Age	-

**Table 2:** Frequency of responses for each questions asked.

	n (%)				
	
Question	Disagree	Somewhat disagree	Neither agree nor disagree	Somewhat agree	Agree
I appreciated the change to more casual dress.	6 (2.6)	14 (6)	15 (6.4)	21 (8.9)	179 (76.2)
Compared to my experience at other programs, the change to more casual dress caused me to worry more about what to wear.	41 (17.7)	18 (7.8)	18 (7.8)	81 (35.1)	73 (31.6)
Compared to my experience at other programs, the change made me feel less comfortable.	134 (58.5)	39 (17)	29 (12.7)	14 (6.1)	13 (5.7)
Compared to my experience at other programs, the change cost me less money.	54 (23.4)	16 (6.9)	97 (42)	11 (4.8)	53 (22.9)
The change in dress expectation lowered Highland in my esteem.	198 (84.6)	14 (6)	14 (6)	6 (2.6)	2 (0.9)
I feel that other programs should change their dress requirements.	21 (9.1)	12 (5.2)	51 (22)	43 (18.5)	105 (45.3)
The lack of uniformity among applicants’ dress made me feel uncomfortable.	144 (62.3)	29 (12.6)	26 (11.3)	26 (11.3)	6 (2.6)
The change reflected the attitudes and demeanor of the Highland Attendings and Residents.	6 (2.6)	2 (0.9)	23 (9.8)	49 (20.9)	154 (65.8)
While interviewing at other programs, other applicants discussed the Highland Dress Expectation (I didn’t bring up the topic).	22 (9.4)	6 (2.6)	11 (4.7)	57 (24.4)	138 (59)
While interviewing at other programs, I mentioned the Highland Dress Expectation initially to other applicants.	58 (24.9)	19 (8.2)	27 (11.6)	59 (25.3)	70 (30)
Compared to my experience at other programs, the change improved my feelings about Highland.	9 (3.9)	4 (1.7)	66 (28.8)	48 (21)	102 (44.5)

**Table 3:** Response frequency to each survey question stratified by gender.*

	n (%)			
	
Question	Disagree	Neither agree nor disagree	Agree	p-value
I appreciated the change to more casual dress.				
Male	14 (10.2)	9 (6.6)	114 (83.2)	0.54
Female	6 (6.2)	6 (6.2)	85 (87.6)	
Compared to my experience at other programs, the change to more casual dress caused me to worry amore about what to wear.				
Male	36 (27.1)	8 (6)	89 (66.9)	0.45
Female	23 (23.7)	10 (10.3)	64 (66)	
Compared to my experience at other programs, the change made me feel less comfortable.				
Male	97 (71.9)	18 (13.3)	20 (14.8)	0.21
Female	75 (80.6)	11 (11.8)	7 (7.5)	
Compared to my experience at other programs, the change cost me less money.				
Male	42 (31.6)	59 (44.4)	32 (24.1)	0.32
Female	28 (28.9)	37 (38.1)	32 (33)	
The change in dress expectation lowered Highland in my esteem.				
Male	122 (89.7)	10 (7.4)	4 (2.9)	0.54
Female	89 (91.8)	4 (4.1)	4 (4.1)	
I feel that other programs should change their dress requirements.				
Male	23 (17)	28 (20.7)	84 (62.2)	0.36
Female	10 (10.4)	22 (22.9)	64 (66.7)	
The lack of uniformity among applicants’ dress made me feel uncomfortable.				
Male	91 (67.4)	19 (14.1)	25 (18.5)	0.008
Female	81 (85.3)	7 (7.4)	7 (7.4)	
The change reflected the attitudes and demeanor of the Highland attendings and residents.				
Male	4 (2.9)	14 (10.3)	118 (86.8)	0.87
Female	4 (4.1)	9 (9.3)	84 (86.6)	
While interviewing at other programs, other applicants discussed the Highland Dress Expectation (I didn’t bring up the topic).				
Male	17 (12.5)	6 (4.4)	113 (83.1)	0.94
Female	11 (11.3)	5 (5.2)	81 (83.5)	
While interviewing at other programs, I mentioned the Highland Dress Expectation initially to other applicants.				
Male	45 (33.6)	15 (11.2)	74 (55.2)	0.94
Female	31 (31.6)	12 (12.2)	55 (56.1)	
Compared to my experience at other programs, the change improved my feelings about Highland.				
Male	7 (5.2)	46 (34.3)	81 (60.4)	0.1
Female	6 (6.4)	20 (21.3)	68 (72.3)	

* “Agree” and “Somewhat agree” responses were consolidated. “Disagree” and “Somewhat disagree” responses were consolidated. Statistical significance did not change when responses were not consolidated.
